# Separating therapeutic efficacy from glucocorticoid side-effects in rodent arthritis using novel, liposomal delivery of dexamethasone phosphate: long-term suppression of arthritis facilitates interval treatment

**DOI:** 10.1186/ar2889

**Published:** 2009-12-15

**Authors:** Una Rauchhaus, Franz Werner Schwaiger, Steffen Panzner

**Affiliations:** 1 Novosom AG, Weinbergweg 22, D-06120 Halle/Saale, Germany; 2 Aurigon Life Science GmbH, Bahnhofstrasse 9-15, D-82327 Tutzing, Germany

## Abstract

**Introduction:**

Glucocorticoids have extensively been used in the treatment of rheumatoid arthritis and other inflammatory diseases. However, their side-effects remain the major limitation in clinical use and an improved therapeutic index is needed.

**Methods:**

Therapeutic efficacy and persistence of free and liposomal dexamethasone phosphate (DXM-P) were determined in mouse collagen-induced arthritis. For regimens with equal therapeutic benefit, the side-effect profiles were analysed over time with respect to collagen breakdown, suppression of the hypothalamus-pituitary-adrenal (HPA) axis, changes in blood glucose levels and the haematological profile. In addition, the presence of drug was monitored in plasma.

**Results:**

Liposomal DXM-P, but not free drug, resulted in a persistent anti-inflammatory effect. Comparable clinical benefit was achieved with a single administration of 4 mg/kg liposomal DXM-P or daily administrations of 1.6 mg/kg free drug for at least 7 days. For the liposomal form, but not for the free form, we observed a limitation of the suppression of the HPA axis in time and an absence of the drug-induced gluconeogenesis.

**Conclusions:**

Liposomal DXM-P, but not free DXM-P, achieves therapeutic persistence in mouse collagen-induced arthritis, which results in drug-free periods of therapeutic benefit. The physical absence of drug after day 2 is associated with a reduction of the typical glucocorticoid side-effects profile. Liposomal DXM-P thereby has an improved therapeutic window.

## Introduction

Glucocorticoids have long been used in the treatment of rheumatoid arthritis, and are an essential part of the first-line anti-inflammatory treatment. Dose escalation and long-term, chronic use of glucocorticoids lead to a number of well-characterized clinical side-effects, however, such as Cushing syndrome [1,2], diabetes [3], or bone demineralization [4], all of which are limiting their therapeutic use.

Strategies for an improvement of the therapeutic index of glucocorticoids focus on the drug molecule itself, its specificity or metabolic conversion [5,6]. Others have investigated selective glucocorticoid receptor agonists to improve the ratio between the therapeutic effect and adverse reaction [7,8]. In addition, the targeted delivery of these drugs using liposomal formulations has been introduced, and a number of studies have demonstrated superior efficacy for water-soluble prednisolone in neutral, polyethylene glycol-modified (PEGylated) liposomes in rheumatoid arthritis animal models and multiple sclerosis [9-12]. A preliminary report on the first clinical use of these formulations confirmed potency and safety in patients [13].

PEGylation on small-sized liposomes is known to minimize uptake into phagocytic cells such as macrophages, which results in extended circulation times, but may be counterindicated for the treatment of inflammatory disorders where macrophages are key producers of proinflammatory cytokines. PEGylation of liposomes has also been associated with the generation of anti-PEG antibodies [14].

We thus developed a non-PEGylated liposomal dexamethasone phosphate (DXM-P), a material that shows cellular uptake in monocytes and macrophages and is devoid of antibody formation even after repeated administration (U Rauchhaus, unpublished results). The material showed a very high accumulation in spleen, whereas drug levels transported into the liver did not exceed peak plasma concentrations. A complete and persistent therapeutic benefit was observed after a single administration [15].

Given the specific distribution of liposomal DXM-P in combination with its therapeutic persistence, we here analyse the potential of this material for a separation of the therapeutic benefit from glucocorticoid-related side-effects. First, a single administration of liposomal DXM-P and daily injections with the free drug were adjusted for equal therapeutic efficacy in mouse collagen-induced arthritis. Second, we characterized the glucocorticoid-related side-effects in both therapeutic modalities. Eventually, the presence of liposomes and dexamethasone were monitored in plasma to correlate presence of the drug with the appearance of its side-effects.

## Materials and methods

### Preparation of liposomal dexamethasone phosphate

Liposomes were prepared from 1,2-dipalmitoyl-*sn*-glycero-3-phosphocholine (DPPC), 1,2-dipalmitoyl-*sn*-glycero-3-(phosphor-*rac*-(1-glcerol)), sodium salt (DPPG), and cholesterol (50:10:40 mol%) using the lipid film extrusion method [16,17]. The lipid film was hydrated with DXM-P (25 mg/ml in PBS, pH 7.5), and the resulting vesicles were extruded through 400 nm membranes. Non-encapsulated DXM-P was removed by gel filtration. The particle size (283 to 310 nm) and polydispersity (<0.3) were determined by dynamic light scattering. The drug/lipid ratio was 40 μg/μmol and the concentration was adjusted to 500 μg DXM-P/ml.

### Animal model of collagen-induced arthritis

All animal studies described here were approved by the Government Commission for Animal Protection.

Arthritis was developed in male DBA/1 mice (age range, 7 to 10 weeks; Taconic Europe, Ry, Denmark) as previously described [18]. Mice were injected with 100 μl type II bovine collagen emulsified in complete Freund's adjuvant (Sigma, Taufkirchen, Germany) on days X1 and X21, and disease progression was monitored in one joint per paw using a pre-defined arthritis index on a scale of 0 (normal) to 4 (ankylosis), resulting in a maximum arthritis index of 16. The incidence of arthritis in the used model protocol was >80%. Animals displaying severe inflammation (arthritis index = 9 to 10) were selected and randomly assigned to the treatment groups (n = 12).

**Figure 1:**
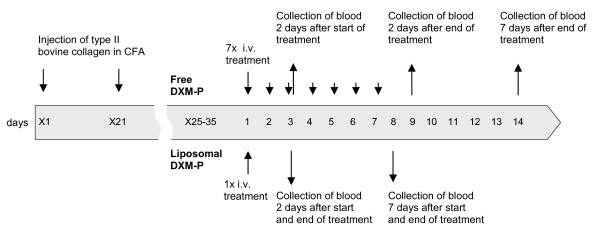
**Study design**. Treatment protocol for administration of free dexamethasone phosphate (DXM-P) and liposomal DXM-P. Blood samples for assessment of the side-effect profile were collected 2 and 7 days after last treatment. An additional blood sample was collected from mice treated with free DXM-P 2 days after start of the treatment. CFA, complete Freund's adjuvant; i.v., intravenous.

Liposomal DXM-P was administered by a single intravenous injection (day 1 of the treatment period), whereas free DXM-P was given daily (days 1 to 7). Single injections with either saline or free DXM-P were used as controls, and the symptoms and side-effects were measured at the time points indicated in Figure 1.

### Analytical measurements

Pyridinoline in urine was measured using a Metra PYD Elisa 8010 at 405 nm (Quidel Corp., San Diego, CA, USA) with creatinine as the internal standard. Blood was analysed on Abbott's CellDyn 3500 (Abbott Diagnostics, Abbott Park, IL, USA). Corticosterone levels in plasma were determined by ELISA (DE3600; R&D Systems, Wiesbaden, Germany) and blood glucose was analysed using Accu-Chek Compact (Roche, Basel, Switzerland).

### Pharmacokinetic analysis of liposomal dexamethasone phosphate

Radiolabelled liposomes (150 μmol lipid, 1.25 MBq ^14^C-DPPC) were processed as above in the presence of 27 MBq ^3^H-Inulin. Obtained particles were 312 nm in size (polydispersity <0.2) and were adjusted with PBS to 47 mM lipid.

For pharmacokinetic assays, 250 g male Wistar rats were given a single intravenous injection of 0.5 ml liposomal preparation. Blood samples of ~0.2 ml were collected and mixed with heparin, and 100 mg of each blood sample was analysed by catalytic oxidation using Ox500 (Zinsser Analytics, Frankfurt, Germany).

### Statistical analysis

The nonparametric Mann-Whitney U test was applied to analyse differences between controls and individual treatments using SPSS 13.0 (SPSS Inc., Chicago, IL, USA). Statistically significant differences were accepted for *P *≤ 0.05.

## Results

### Efficacy of liposomal and free dexamethasone phosphate

**Figure 2:**
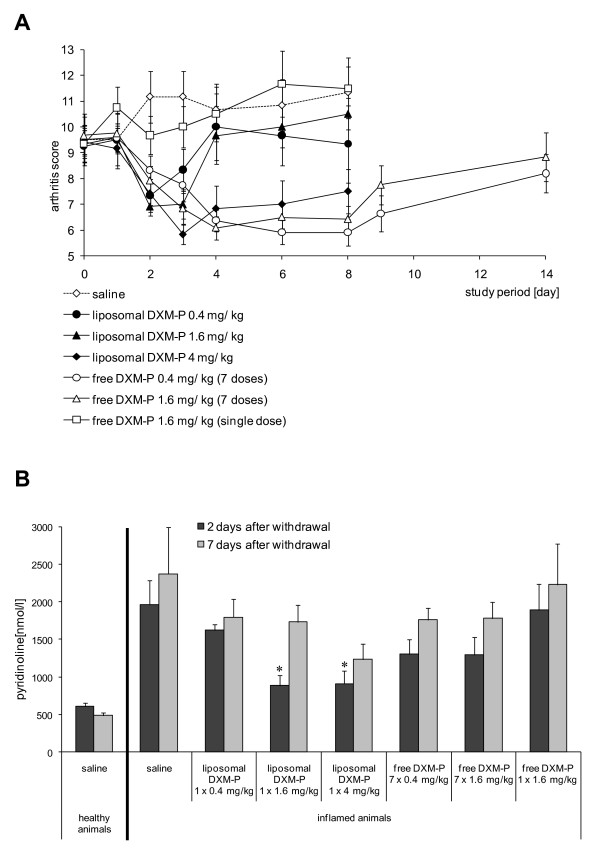
**Therapeutic efficacy and persistence of liposomal dexamethasone phosphate**. **(a) **Joint swelling was assessed by clinical scoring after a single injection of liposomal dexamethasone phosphate (DXM-P) (0.4 mg/kg, 1.6 mg/kg or 4 mg/kg) or after seven daily injections of free DXM-P (0.4 and 1.6 mg/kg). A single dose of free DXM-P at 1.6 mg/kg is also shown for comparison. Saline injections were used as controls. n = 12 for all groups; means ± standard error of the mean. **(b) **Urine pyridinoline levels 2 and 7 days after cessation of the treatment. *P ≤ 0.05 Mann-Whitney U test versus saline-treated inflamed animals. n = 6 for all groups; means ± standard error of the mean.

Collagen-induced arthritis was established in mice, and groups of 12 mice were treated with liposomal DXM-P or free DXM-P, respectively. Single administrations of 0.4 to 4 mg/kg liposomal DXM-P generated a rapid and substantial reduction of the paw swelling. In addition, the highest dose resulted in a persistent remission of arthritis for at least 7 days. In contrast, a single dose of 1.6 mg/kg free DXM-P was ineffective in this model, and daily injections of 0.4 or 1.6 mg/kg free drug were required to suppress paw swelling over the treatment period of 7 days. The onset of therapeutic improvement was substantially delayed when using daily injections of 0.4 mg/kg free DXM-P (Figure 2a).

We also determined concentrations of urinary pyridinoline to monitor the arthritis-related degradation of bone and cartilage. Mature collagen chains in these tissues are connected by 3-hydroxypyridinium crosslinks, which become discharged and excreted as pyridinoline upon disintegration of collagen [19].

Levels of urinary pyridinoline are about 500 nM in healthy animals and 2,000 nM in arthritic animals. Treatment with moderate or high doses of liposomal DXM-P resulted in a reduction well below 1,000 nM on day 2. In addition, the single administration of 4 mg/kg liposomal DXM-P resulted in a sustained reduction of this marker through to day 7. Daily use of free DXM-P, although effective in the reduction of paw volumes and arthritic scores, did not afford a significant inhibition of the collagen breakdown (Figure 2b).

### Side-effect profile of liposomal dexamethasone phosphate

We next set out to directly compare glucocorticoid-mediated side-effects for both treatment modalities. In the liposome arm of the study, three groups of mice (n = 12) received a single dose of 0.4, 1.6 or 4 mg/kg liposomal DXM-P on day 1 and the side-effects were monitored 2 or 7 days later (n = 6 each).

In a second arm of the study, two groups of mice were treated with daily injections of 0.4 or 1.6 mg/kg free DXM-P (n = 12). Side-effects were monitored as above; that is, 2 or 7 days after cessation of treatment (n = 6 each).

For corticosterone, base levels for all animals were taken on day (X - 5) and all changes were expressed as animal-specific values relative to this starting point (n = 12). Additional data were obtained on day 3 for groups receiving daily free DXM-P (n = 12) to facilitate a direct comparison between groups at this point in time (Figure 1).

Control groups (n = 12) received single administrations of 1.6 mg/kg free drug or saline and were monitored 2 and 7 days later (n = 6 each).

#### Blood corticosterone

Glucocorticoids inhibit the release of adrenocorticotropic hormone from the pituitary gland, leading to a suppression of corticosterone production in the adrenal glands [20]. On the contrary, increased corticosterone levels are caused by the inflammation process itself [21]. We therefore analysed the response of corticosterone to liposomal DXM-P or free DXM-P in healthy and inflamed animals.

**Figure 3:**
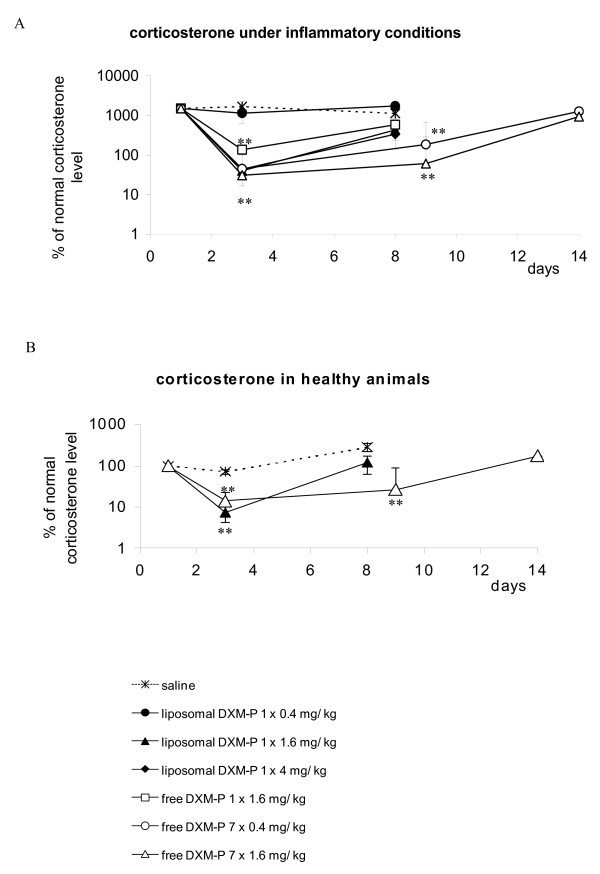
**Impact of liposomal and free dexamethasone phosphate on corticosterone**. Corticosterone levels in the blood of **(a) **arthritic animals or **(b) **healthy animals were measured following treatment with liposomal dexamethasone phosphate (DXM-P), free DXM-P or saline. **P ≤ 0.01 Mann-Whitney U test versus saline. *n *≥ 6 for all groups; mean ± standard error of the mean.

A low dose of 0.4 mg/kg liposomal DXM-P did not change the level of corticosterone in arthritic mice, while improving the clinical score on days 2 and 3. In contrast, a single administration of 1.6 mg/kg free DXM-P resulted in suppression of corticosterone at day 3 without delivering such therapeutic benefit (*P *< 0.01 vs. saline) (Figure 3a).

Use of 1.6 or 4 mg/kg liposomal DXM-P also reduced serum corticosterone levels (*P *≤ 0.01 vs. saline); however, this effect was transient since corticosterone production recovered by day 9. The normalization was complete in healthy animals, but not in inflamed mice. We thus attribute this incomplete reversion to the therapeutic benefit of the liposomal drug rather than to a long-term suppression of corticosterone (Figure 3a, b).

Animals treated with free DXM-P continued to display a significantly suppressed corticosterone level over the entire treatment period. As with the liposomal form, these values normalized after cessation of treatment.

#### Blood glucose

**Figure 4:**
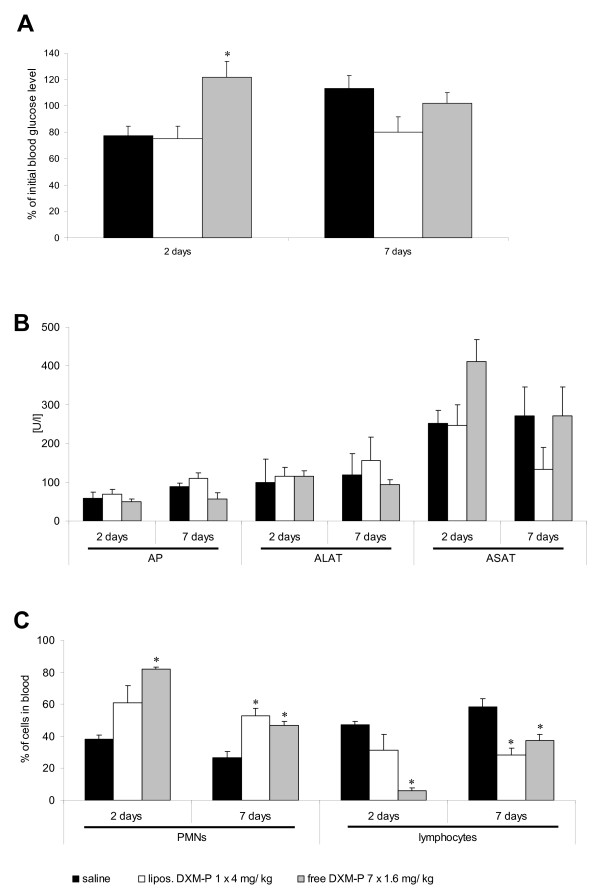
**Effects of liposomal and free dexamethasone phosphate on blood glucose, liver enzymes and haematology**. **(a) **Glucose levels as a percentage of the initial blood value in arthritic animals following treatment with liposomal dexamethasone phosphate (DXM-P), free DXM-P, or saline. **(b) **Values of liver enzymes alkaline phosphatase (AP), alanine aminotransferase (ALAT) and aspartate aminotransferase (ASAT) after treatment with liposomal DXM-P, free DXM-P, or saline. **(c) **Percentage of polymorphonuclear neutrophilic leukocytes (PMNs) and lymphocytes in the blood of arthritic animals following treatment with liposomal DXM-P, free DXM-P, or saline. *P ≤ 0.05 Mann-Whitney U test versus saline-treated inflamed animals. n = 6 for all groups; mean ± standard error of the mean.

Daily administration of therapeutic amounts of free DXM-P led to a significant increase of the blood glucose, with levels persisting for at least 2 days after the last treatment - which is day 9 under this regimen. In contrast, a single injection of 4 mg/kg liposomal DXM-P did not alter blood glucose levels 2 days after the injection, which is day 3 in this group. Independent of the respective treatments, no significant alterations of the blood glucose levels were monitored at day 7 after the last administration of drug (Figure 4a).

#### Liver enzymes

A slight, but nonsignificant, elevation of liver aspartate aminotransferase was connected to the use of free DXM-P, but not of liposomal DXM-P. Any such elevation was no longer detectable at day 7 after the termination of treatment (Figure 4b).

#### Haematology

Glucocorticoids are known to induce neutrophilia by stimulating the migration of immature polymorphonuclear neutrophils from bone marrow into the circulation [22,23].

We confirmed this knowledge and observed a neutrophilia upon daily administrations of 1.6 mg/kg free DXM-P. Treatment with 4 mg/kg liposomal DXM-P resulted in a milder and delayed increase of the number of neutrophils (Figure 4c), which became significant only at day 7 after the cessation of treatment.

The use of free DXM-P is lymphopenic; a significantly reduced number of lymphocytes was thus observed during the treatment with daily doses of 1.6 mg/kg free drug. In contrast, treatment with 4 mg/kg liposomal DXM-P caused only a mild lymphopenia (Figure 4c). Also, the lymphopenia became significant only at the later time point. The reasons for these delayed effects of the liposomal drug on neutrophils and lymphocytes are not known and require further investigation. No other haematological alterations were observed in any of the groups.

#### Body weight

A reduction in body weight is a common phenomenon of the use of glucocorticoids in rodents [24]. In the model described here, daily injections of 1.6 mg/kg free DXM-P reduced the body weight at day 8 by 10%. The same reduction was observed for a single injection of 4 mg/kg liposomal DXM-P. The lower doses of 1.6 or 0.4 mg/kg of the liposomal form resulted in less than 4% reduction of body weight.

### Presence of free and liposomal dexamethasone phosphate in plasma

We eventually analysed the pharmacokinetic of free and liposomal DXM-P to correlate the presence of drug with the observed side-effects. This analysis was performed in rats due to the technical limitations in mice.

**Figure 5:**
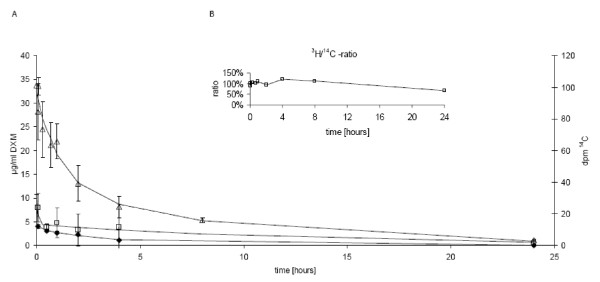
**Pharmacokinetics of liposomal and free dexamethasone phosphate in rats**. **(a) **A dose of 4 mg/kg liposomal dexamethasone phosphate (DXM-P) (open squares) or free DXM-P (diamonds) was injected intravenously into rats, and the appearance of the converted dexamethasone (DXM) was monitored in plasma. In a separate experiment, radiolabelled liposomal DXM-P (triangles) was injected into rats and their pharmacokinetics was followed using ^14^C-1,2-dipalmitoyl-*sn*-glycero-3-phosphocholine (DPPC). **(b) **Integrity of liposomal DXM-P. Radiolabelled liposomal DXM-P was injected as in (a) and the additional aqueous phase marker ^3^H-inuline was analysed. The resulting ^3^H/^14^C ratio was plotted with respect to the initial ratio. The constant ratio indicates high carrier integrity in the circulation. Error bars indicate standard deviations.

Free DXM-P is rapidly converted into dexamethasone immediately after injection, with a half-life of 1.3 minutes [25]. Dexamethasone itself has a longer circulating half-life of 150 minutes (Figure 5a, black diamonds).

Liposomal DXM-P circulates in an intact form, as demonstrated by the constant sequestration of the aqueous phase marker ^3^H-inulin (an uncharged polyfructose; molecular weight, ~5,000 Da) with respect to the lipid membrane marker ^14^C-DPPC (Figure 5b) in a labelled probe of liposomal DXM-P. The elimination of liposomes from the circulation followed a two-phase elimination profile: 60% of the particles disappear with a half-life of 44 minutes; the remainder circulates with a half-life of 370 minutes.

A first portion of dexamethasone appeared shortly after the injection (Figure 5a, squares), indicating a small fraction of free DXM-P or drug associated with the outer layer of the liposomes. The bulk of dexamethasone appeared in plasma slowly and left the compartment with a terminal half-life of 520 minutes.

Both administrations of free DXM-P or liposomal DXM-P do not yield persistent drug levels in plasma. Only low levels of the drug are physically present at 24 hours, and certainly at 48 hours, after the last administration.

## Discussion

In the present study, we used a mouse model of rheumatoid arthritis to compare different treatment regimes for DXM-P in its liposomal and free forms with respect to clinical outcome and to the side-effects profile.

Liposomal DXM-P displayed a persistent therapeutic effect, as a single injection of liposomal DXM-P at 4 mg/kg suppressed established arthritis for at least 7 days. In contrast, treatment with free DXM-P required daily administrations of 1.6 mg/kg to achieve a continued suppression of the paw swelling; a single treatment resulted in no detectable therapeutic benefit.

The liposomal formulation also afforded significant reduction of pyridinoline, a marker for bone and cartilage degradation. Such reduction of pyridinoline provides additional proof of the strong therapeutic activity of liposomal DXM-P and correlates to the reduced joint destruction observed for liposomal DXM-P, but not free DXM-P as reported earlier [15].

The combined efficacy data provided herein support comparable therapeutic benefit for a single administration of 4 mg/kg liposomal DMX-P and daily administrations of at least 1.6 mg/kg free glucocorticoid. This regimen is based on the limited reduction of pyridinoline even after seven doses of 1.6 mg/kg free drug, on the slower remission caused by the free drug and on the absence of any therapeutic improvement after a single administration of 1.6 mg/kg free DXM-P.

Glucocorticoids interfere with the hypothalamus-pituitary-adrenal (HPA) axis, in that they compete with the natural ligand corticosterone. The clinical manifestation of this interference is Cushing syndrome, which is one of leading dose-limiting factors for glucocorticoids [1,26].

Free DXM-P as well as higher doses of liposomal DXM-P did show an adverse impact on the HPA axis on day 3. The appearance of this corticosterone modulation is consistent with the presence of residual amounts of DXM-P in the circulation at this point in time. Our data, however, support a view wherein the suppression of the HPA axis is substantially shorter than the persistent therapeutic effect achieved with liposomal DXM-P. Persistence may thus facilitate a separation between therapeutic benefit and side-effects, at least in time.

In contrast to the liposomal dosage form, the free drug does not allow such separation between HPA suppression and therapeutic effect. Even worse, we found a sensitive suppression of the HPA axis using a single, therapeutically insufficient, administration of free DXM-P. In contrast, no such suppression was monitored after treatment with a single dose of liposomal DXM-P at 0.4 mg/kg, which was therapeutically active. This finding provides a separation of the therapeutic benefit from related side-effects using substantially reduced amounts of drug.

Liposomal DXM-P did not induce hyperglycaemia across the entire dose range, whereas daily treatments with the free drug significantly increased blood glucose levels. This observation is in line with the very low hepatic accumulation of the liposomal drug that does not exceed peak plasma concentrations [15]. In addition, any such uptake does mainly occur in the phagocytic Kupffer cells - but not in hepatocytes, the main cell type associated with gluconeogenesis (U Rauchhaus, unpublished observations). We relate this to the large size of the liposomes of about 300 nm, which is well above the exclusion limit for the liver endothelium of about 100 nm [27].

Liposomal DXM-P also has a reduced impact on the complete blood count. Exposure to free DXM-P resulted in significant, but transient, neutrophilia and lymphopenia. The effect of the liposomal DXM-P was modest and no significant changes were observed on day 2. Long-term alterations in lymphocytes and neutrophils, although significant by day 7, had small amplitude. Although a mild lymphopenia was observed in the collagen-induced arthritis model used here, the impact of liposomal DXM-P on immune organs is generally quite modest. We observed no pathological alterations in the spleen after administration of up to 30 mg/kg liposomal DXM-P into healthy rats. Occasional hypotrophism of the thymus was observed with doses of 10 mg/kg or more.

## Conclusions

Glucocorticoids belong to the basic therapeutic arsenal in the field of inflammatory and autoimmune diseases. Being powerful drugs, their clinical acceptance is mainly limited by side-effects.

Using a liposomal dosage form of DXM-P we here observe a separation between the therapeutic benefit and side-effects. We attribute these improvements in the side-effect profile to a targeted, cellular delivery of the liposomal dosage form to cells and organs of the immune system, mainly the spleen, which we hypothesize to drive the therapeutic persistence [15].

At the same time, the liposomal form excludes the drug from reaching unwanted sites. This exclusion became most apparent when a small, yet therapeutically active, dose of liposomal DXM-P had no impact on the HPA axis, whereas even subtherapeutic amounts of the free drug reduced the corticosterone production.

Of note, the increased therapeutic potency of liposomal DXM-P resulted also in a significant reduction of urinary pyridinoline, indicating an inhibition of the ongoing, inflammation-mediated breakdown of collagen. If translated into clinical practice, such a feature would offer a window for regeneration of the inflamed site, thus establishing a disease-modifying quality for glucocorticoids.

Future developmental work should address the therapeutic persistence, in particular. The intermittent use of liposomal DXM-P has the potential for an improved therapeutic index in that it combines an immediate but lasting therapeutic benefit with side-effects that are restricted to the actual time of treatment.

## Abbreviations

DPPC: 1,2-dipalmitoyl-*sn*-glycero-3-phosphocholine; DXM-P: dexamethasone phosphate; ELISA: enzyme-linked immunosorbent assay; HPA: hypothalamus-pituitary-adrenal; PBS: phosphate-buffered saline; PEG: polyethylene glycol.

## Competing interests

SP and UR are employees of Novosom AG - Novosom AG funded this research. SP is founder and shareholder of the Novosom AG - Novosom AG holds or has applied for patents relating to the content of the manuscript (liposomal glucocorticoids). No payments have been made to the authors.

## Authors' contributions

UR and SP developed the liposomes used in this publication; both conceived and coordinated the studies, interpreted the data and prepared the manuscript. FWS performed experimental work in the collagen-induced arthritis model.

## References

[B1] Hopkins RL Leinung MC Exogenous Cushing's syndrome and glucocorticoid withdrawal Endocrinol Metab Clin North Am 2005 34 371 384 ix 10.1016/j.ecl.2005.01.013 15850848

[B2] Saklatvala J Glucocorticoids: do we know how they work? Arthritis Res 2002 4 146 150 10.1186/ar398 12010562 PMC128923

[B3] Wang M The role of glucocorticoid action in the pathophysiology of the metabolic syndrome Nutr Metab (Lond) 2005 2 3 10.1186/1743-7075-2-3 15689240 PMC548667

[B4] Manolagas SC Weinstein RS New developments in the pathogenesis and treatment of steroid-induced osteoporosis J Bone Miner Res 1999 14 1061 1066 10.1359/jbmr.1999.14.7.1061 10404005

[B5] Buttgereit F Wehling M Burmester GR A new hypothesis of modular glucocorticoid actions: steroid treatment of rheumatic diseases revisited Arthritis Rheum 1998 41 761 767 10.1002/1529-0131(199805)41:5<761::AID-ART2>3.0.CO;2-M 9588727

[B6] Buttgereit F Straub RH Wehling M Burmester GR Glucocorticoids in the treatment of rheumatic diseases: an update on the mechanisms of action Arthritis Rheum 2004 50 3408 3417 10.1002/art.20583 15529366

[B7] Song IH Gold R Straub RH Burmester GR Buttgereit F New glucocorticoids on the horizon: repress, don't activate! J Rheumatol 2005 32 1199 1207 16041872

[B8] Schacke H Hennekes H Schottelius A Jaroch S Lehmann M Schmees N Rehwinkel H Asadullah K SEGRAs: a novel class of anti-inflammatory compounds Ernst Schering Res Found Workshop 2002 40 357 371 12355726 10.1007/978-3-662-04660-9_20

[B9] Avnir Y Ulmansky R Wasserman V Even-Chen S Broyer M Barenholz Y Naparstek Y Amphipathic weak acid glucocorticoid prodrugs remote-loaded into sterically stabilized nanoliposomes evaluated in arthritic rats and in a Beagle dog: a novel approach to treating autoimmune arthritis Arthritis Rheum 2008 58 119 129 10.1002/art.23230 18163482

[B10] Metselaar JM Wauben MH Wagenaar-Hilbers JP Boerman OC Storm G Complete remission of experimental arthritis by joint targeting of glucocorticoids with long-circulating liposomes Arthritis Rheum 2003 48 2059 2066 10.1002/art.11140 12847701

[B11] Metselaar JM Berg WB van den Holthuysen AE Wauben MH Storm G van Lent PL Liposomal targeting of glucocorticoids to synovial lining cells strongly increases therapeutic benefit in collagen type II arthritis Ann Rheum Dis 2004 63 348 353 10.1136/ard.2003.009944 15020326 PMC1754935

[B12] Schmidt J Metselaar JM Wauben MH Toyka KV Storm G Gold R Drug targeting by long-circulating liposomal glucocorticosteroids increases therapeutic efficacy in a model of multiple sclerosis Brain 2003 126 1895 1904 10.1093/brain/awg176 12805101

[B13] Barrera P Mulder S Smetsers A Storm G Beijnen J Metselaar J van Riel P Long-circulating liposomal prednisolone versus pulse intramuscular methylprednisolone in patients with active rheumatoid arthritis [abstract] Proceedings of the American College of Rheumatology (ACR) Scientific Meeting; Oct 24-29, 2008; San Francisco. Hall A, poster board 453 2008

[B14] Ishida T Ichihara M Wang X Yamamoto K Kimura J Majima E Kiwada H Injection of PEGylated liposomes in rats elicits PEG-specific IgM, which is responsible for rapid elimination of a second dose of PEGylated liposomes J Control Release 2006 112 15 25 10.1016/j.jconrel.2006.01.005 16515818

[B15] Rauchhaus U Kinne RW Pohlers D Wiegand S Wolfert A Gajda M Brauer R Panzner S Targeted delivery of liposomal dexamethasone phosphate to the spleen provides a persistent therapeutic effect in rat antigen-induced arthritis Ann Rheum Dis 2009 68 1933 1934 10.1136/ard.2009.108985 19910302

[B16] Olson F Hunt CA Szoka FC Vail WJ Papahadjopoulos D Preparation of liposomes of defined size distribution by extrusion through polycarbonate membranes Biochim Biophys Acta 1979 557 9 23 10.1016/0005-2736(79)90085-3 95096

[B17] Szoka F Jr Papahadjopoulos D Comparative properties and methods of preparation of lipid vesicles (liposomes) Annu Rev Biophys Bioeng 1980 9 467 508 10.1146/annurev.bb.09.060180.002343 6994593

[B18] Durie FH Fava RA Noelle RJ Collagen-induced arthritis as a model of rheumatoid arthritis Clin Immunol Immunopathol 1994 73 11 18 10.1006/clin.1994.1164 7923907

[B19] Seibel MJ Woitge H Scheidt-Nave C Leidig-Bruckner G Duncan A Nicol P Ziegler R Robins SP Urinary hydroxypyridinium crosslinks of collagen in population-based screening for overt vertebral osteoporosis: results of a pilot study J Bone Miner Res 1994 9 1433 1440 7817828 10.1002/jbmr.5650090916

[B20] Kuperman H Damiani D Chrousos GP Dichtchekenian V Manna TD Filho VO Setian N Evaluation of the hypothalamic-pituitary-adrenal axis in children with leukemia before and after 6 weeks of high-dose glucocorticoid therapy J Clin Endocrinol Metab 2001 86 2993 2996 10.1210/jc.86.7.2993 11443157

[B21] Gaillard RC Interaction between the hypothalamo-pituitary-adrenal axis and the immunological system Ann Endocrinol (Paris) 2001 62 155 163 11353887

[B22] Claman HN How corticosteroids work J Allergy Clin Immunol 1975 55 145 151 10.1016/0091-6749(75)90010-X 803519

[B23] Peng CT Lin HC Lin YJ Tsai CH Yeh TF Early dexamethasone therapy and blood cell count in preterm infants Pediatrics 1999 104 476 481 10.1542/peds.104.3.476 10469772

[B24] Rooman R Koster G Bloemen R Gresnigt R van Buul-Offers SC The effect of dexamethasone on body and organ growth of normal and IGF-II-transgenic mice J Endocrinol 1999 163 543 552 10.1677/joe.0.1630543 10588828

[B25] Rohdewald P Mollmann H Barth J Rehder J Derendorf H Pharmacokinetics of dexamethasone and its phosphate ester Biopharm Drug Dispos 1987 8 205 212 10.1002/bdd.2510080302 3593899

[B26] Hatz H Glukokortikoide. Immunologische Grundlagen, Pharmakologie und Therapierichtlinien 2005 2 Stuttgart: Wissenschaftliche Verlagsgesellschaft mbH

[B27] Braet F Wisse E Structural and functional aspects of liver sinusoidal endothelial cell fenestrae: a review Comp Hepatol 2002 1 1 10.1186/1476-5926-1-1 12437787 PMC131011

